# The Effects of Acute Cardiovascular Exercise on Memory and Its Associations With Exercise-Induced Increases in Neurotrophic Factors

**DOI:** 10.3389/fnagi.2021.750401

**Published:** 2021-11-08

**Authors:** Laura A. Kuhne, Anna-Maria Ksiezarczyk, Klaus-Michael Braumann, Rüdiger Reer, Thomas Jacobs, Brigitte Röder, Kirsten Hötting

**Affiliations:** ^1^Biological Psychology and Neuropsychology, Institute of Psychology, University of Hamburg, Hamburg, Germany; ^2^Sports and Exercise Medicine, Institute of Human Movement Science, University of Hamburg, Hamburg, Germany; ^3^Protozoa Immunology, Bernhard Nocht Institute for Tropical Medicine, Hamburg, Germany

**Keywords:** learning, memory, physical exercise, neurotrophic factors, BDNF, VEGF

## Abstract

Due to increasing life expectancy, low-cost interventions to counteract age-related memory impairment have gained popularity. Physical activity has been shown to positively affect memory and hippocampal plasticity in rodents and humans. These effects have been proposed to be mediated by the release of neurotrophic factors. However, studies examining the effects of a single cardiovascular exercise session on human memory have yielded conflicting results. Moreover, it remains unclear whether exercise-induced memory enhancements are related to changes in peripheral neurotrophic factor concentrations. The present study tested whether one bout of cardiovascular exercise during an early phase of memory consolidation, compared to one bout of stretching and toning, positively affected memory. Furthermore, it was analyzed whether exercise-induced changes in the brain-derived neurotrophic factor (BDNF) and vascular endothelial growth factor (VEGF) were related to memory enhancement after a single bout of physical exercise. Fifty healthy participants (20–40 years) were randomly assigned to either a cycling group (BIKE) or a stretching and toning group (STRETCH). Participants performed an implicit vocabulary learning task which was immediately followed by physical exercise. Memory for the learned vocabulary was tested 1–2 weeks later. To measure exercise-induced changes in serum neurotrophic factor levels, blood samples were collected at rest (baseline) and immediately after the exercise session. Results did not show a significant difference in memory between the BIKE group and the STRETCH group. However, in the BIKE group, a larger increase in BDNF and VEGF levels was observed than in the STRETCH group. Moreover, the increase in BDNF and memory performance tended to be positively related in the BIKE group. We speculate that the correlation between exercise-increased BDNF levels and memory in the cycling group may indicate an involvement of BDNF in mediating memory processes after acute cardiovascular exercise.

## Introduction

Life expectancy has been increasing over the past decades (United Nations, 2013). As a result, a growing number of individuals are subject to age-related cognitive decline (Prince et al., 2015). Executive functions, processing speed, and memory are typically mostly affected (Hedden and Gabrieli, 2004). Pathological progression of memory impairments resulting in, for example, Alzheimer’s disease, have been reported to be one of the greatest worries of the population about living beyond the age of 75 (Anderson and McConnell, 2007). Thus, there is growing interest in interventions that potentially counteract age-related memory decline.

Regular cardiovascular exercise has been reported to positively influence memory and to induce structural and functional changes in brain regions associated with memory, e.g., the hippocampus. Several weeks or months of regular cardiovascular training were shown to increase performance in a face-name matching task (Griffin et al., 2011), visuospatial short-term memory (Stroth et al., 2009), and immediate and delayed memory for wordlists (Chapman et al., 2013). The increase in cardiovascular fitness after physical exercise training has been found to correlate positively with improvements in episodic memory (Hötting et al., 2012). Moreover, increased hippocampus volume (Erickson et al., 2011; Niemann et al., 2014; Thomas A. G. et al., 2016) and increased hippocampal cerebral blood volume (Pereira et al., 2007) have been reported after a period of regular cardiovascular training. However, evidence in humans concerning the effects of long-term aerobic exercise on memory and associated brain structures in the medial temporal lobe is contradictory. Some studies reported positive effects of an exercise intervention on memory (Stroth et al., 2009; Griffin et al., 2011), while others failed to find a difference in memory between an aerobic exercise group and a control group (Gourgouvelis et al., 2018). In a meta-analysis, Roig et al. (2013) concluded that the positive effects of regular cardiovascular training on long-term memory were not reliably found. Participants’ age, training intensity, training duration, and the type of memory tested have been discussed to in part account for the differing results. A better understanding of possible mediators underlying the effects of cardiovascular exercise on memory may resolve some of the observed inconsistencies and shed light on why some training studies yield more consistent exercise-induced memory effects than others.

Similar to chronic cardiovascular exercise, results for single bouts of exercise on memory functions are inconsistent. On the one hand, a single bout of cardiovascular exercise has been shown to positively affect memory (Roig et al., 2013; Bosch et al., 2017b; Dal Maso et al., 2018). On the other hand, other researchers did not find an acute effect of exercise on memory (Hopkins et al., 2012; Basso et al., 2015), or reported enhancements of specific aspects of memory (Coles and Tomporowski, 2008; Suwabe et al., 2017). Inconsistencies in results from human studies are potentially caused by variations in the type of memory tested, exercise intensity, as well as the timing of the exercise session in relation to memory encoding (Roig et al., 2013).

To date, only a few studies have tested the effects of cardiovascular exercise on memory after encoding, i.e., during memory consolidation, and their results have been inconclusive. Beneficial outcomes have been reported for procedural memory when retention was measured 24 h (Roig et al., 2012; Dal Maso et al., 2018) or several days after encoding (Roig et al., 2012; McNerney and Radvansky, 2015). Additionally, an EEG study revealed that better skill retention after exercising was associated with greater beta-band event-related desynchronization in sensorimotor areas, indicating that exercise may improve motor memory by modulating neuronal processing in motor cortices in the early stages of memory consolidation (Dal Maso et al., 2018). However, the effects of cardiovascular exercise after encoding on declarative memory are more equivocal. In one study, participants performed an aerobic exercise session either before or after exposure to a word list which they were instructed to memorize. Memory was tested 60 min and 24 h after learning (Labban and Etnier, 2018). At neither time point did the results indicate a beneficial effect of exercising compared to a no-exercising condition during the consolidation phase. In another study, 6 min of either cycling or relaxing after encoding of emotional images resulted in better memory performance in the cycling group when assessed 60 min after exercise, and thus suggested a beneficial effect of cardiovascular exercise on memory consolidation (Segal et al., 2012). Other researchers compared the effects of 30 min of cycling at low or high intensity to the effects of relaxing after vocabulary learning (Hötting et al., 2016). Cycling after encoding did not enhance the absolute number of recalled words tested 60 min and 24 h after learning. However, participants who exercised at high intensity showed less forgetting between the 60-min and 24-h measurements than participants in the relaxation group. These findings suggested a benefit of cardiovascular exercise directly after encoding on memory consolidation. van Dongen et al. (2016) demonstrated that cycling with a 4-h delay after encoding revealed better performance in a hippocampus-dependent picture-location association task, compared to a group cycling directly after encoding, and a no-exercise control group. In the same study, fMRI data revealed that participants who cycled 4 h after learning showed more distinctive hippocampal representations for the learned associations during retrieval, compared to participants of the other two groups. Furthermore, higher hippocampal pattern similarity correlated with better memory retention across participants. These data suggested that cardiovascular exercise might enhance later stages of memory consolidation more than early phases. In summary, current data on the effects of cardiovascular exercise after encoding on memory performance are ambiguous. Most studies reporting beneficial effects of a single bout of exercise on memory consolidation used sensorimotor tasks. Studies assessing declarative memory are rare and their findings are inconsistent.

Cardiovascular exercise has been proposed to affect memory by increasing levels of neurochemical substances, such as hormones, neurotransmitters, and neurotrophic factors known to be involved in the formation of memories on the neuronal level (Basso and Suzuki, 2017). It has been suggested that early stages of memory consolidation, when the memory trace has most likely not yet reached a stable state, may be particularly susceptible to external influences, such as exercise (Nader and Hardt, 2009). Accordingly, research in both rodents and humans has shown that early memory traces can be altered by stress, physical activity, and pharmacological treatments (McGaugh, 1966; Siette et al., 2014; Vogel et al., 2016).

One of the neurochemical pathways postulated to mediate the effects of cardiovascular exercise on memory involves the exercise-induced alteration of neurotrophic factor levels, such as brain-derived neurotrophic factor (BDNF), vascular endothelial growth factor (VEGF), and insulin-like growth factor 1 (IGF-1; Cotman et al., 2007). In rodents, BDNF, VEGF, and IGF-1 have been shown to be elevated after wheel running (Cetinkaya et al., 2013; Uysal et al., 2015). Moreover, they have been shown to be involved in processes underlying memory formation (Cotman et al., 2007; Voss et al., 2013). BDNF, for instance, is involved in the morphological changes of dendritic spines, long-term potentiation (LTP), and increases neurogenesis by promoting cell survival and proliferation (Bekinschtein et al., 2014; Miranda et al., 2019). LTP is one of the primary mechanisms of synaptic plasticity underlying memory and learning processes. As a key regulator of LTP, BDNF has become a particularly prominent target of research (Miranda et al., 2019). VEGF has been proposed to modulate memory by being involved in angiogenesis and neurogenesis (Fabel et al., 2003; Greenberg and Jin, 2005). IGF-1 is thought to have a neuroprotective function, to play a role in neurogenesis, angiogenesis, synaptic plasticity, and to interact with VEGF to enhance neurogenesis after exercise (Cotman et al., 2007; Fernandez and Torres-Alemán, 2012). Inhibiting BDNF and VEGF action in rodents has been shown to prevent running-induced memory benefits (Vaynman et al., 2004) and neurogenesis (Fabel et al., 2003), respectively. While blocking of IGF-1 activity did not alter the positive effect of exercise on learning, it prevented exercise-induced memory benefits when tested 2 days after learning (Ding et al., 2006). In addition, exercise-induced increases in BDNF, VEGF, and IGF-1 have been shown to correlate with improved spatial learning and memory (Cetinkaya et al., 2013; Uysal et al., 2015). Thus, the results of rodent research suggested that BDNF, VEGF, and IGF-1 mediate the beneficial effects of physical activity on neuroplasticity and memory.

In contrast to rodents, neurotrophic factors cannot be measured directly in the living human brain. Measures are usually taken from blood serum or plasma. In some studies, BDNF, VEGF, and IGF-1 have been shown to cross the blood-brain barrier, indicating a transferability from peripheral to central levels (Pan et al., 1998; Nishijima et al., 2010; Rich et al., 2017; but see Lanz et al., 2012). In humans, a single bout of moderate to intense aerobic exercise has been shown to transiently increase BDNF levels (Ferris et al., 2007; Winter et al., 2007; Hötting et al., 2016; Tsai et al., 2018). In contrast, the evidence for changes in VEGF and IGF-1 levels after cardiovascular exercise in humans is limited in number, and results are equivocal (Griffin et al., 2011; Skriver et al., 2014). Only a few studies reported increases in VEGF and IGF-1 after acute exercise (Kraemer et al., 2004; Kraus et al., 2004; Skriver et al., 2014; Tsai et al., 2018). Associations between the change in neurotrophic factor levels and memory, which might indicate an involvement of neurotrophic factors in memory processes, have not reliably been shown in human studies. Some researchers have demonstrated that increased BDNF levels after exercising correlate with vocabulary learning (Winter et al., 2007) and motor memory (Skriver et al., 2014), while others did not find a relationship with performance in episodic memory tasks (Schmidt-Kassow et al., 2014; Etnier et al., 2016). So far, human studies have failed to find correlations between acute increases in VEGF and IGF-1 after exercising and memory measures (Skriver et al., 2014; Tsai et al., 2018). Hence, a relationship between changes in neurotrophic factors and exercise-induced memory improvements in humans has not been reliably demonstrated to date.

The goal of the present study was to test whether one bout of cardiovascular exercise after encoding had beneficial effects on hippocampus-dependent memory compared to a non-cardiovascular exercise bout after the same memory task. Moreover, we examined whether memory effects were related to exercise-induced changes in neurotrophic factor levels. Young participants were randomized to either a cycling (BIKE) or a stretching and toning training (STRETCH). Immediately after the encoding of an artificial vocabulary, that is during an early stage of memory consolidation, participants engaged in a single bout of physical exercise. Memory was assessed 1–2 weeks after the initial vocabulary acquisition. Blood samples were taken at rest (baseline) and directly after an acute bout of physical exercise to measure serum levels of BDNF and VEGF. We hypothesized that cardiovascular exercise after encoding would result in enhanced memory for the encoded vocabulary compared to stretching and toning. Moreover, it was assumed that cardiovascular exercise, compared to stretching and toning, increased BDNF and VEGF levels. Exercise-induced increases in both neurotrophic factor levels were thought to positively correlate with memory performance.

## Materials and Methods

### Participants

Participants of this study took part in a larger randomized training study that spanned 10 weeks of treatment with repeated learning sessions followed by physical exercise. *A priori* sample size calculation was based on the planned analyses of the larger study project. Here we focus on the acute effects of a single exercise session on learning and memory. Results on the chronic effects of 10 weeks of training will be reported elsewhere.

**Figure 1:**
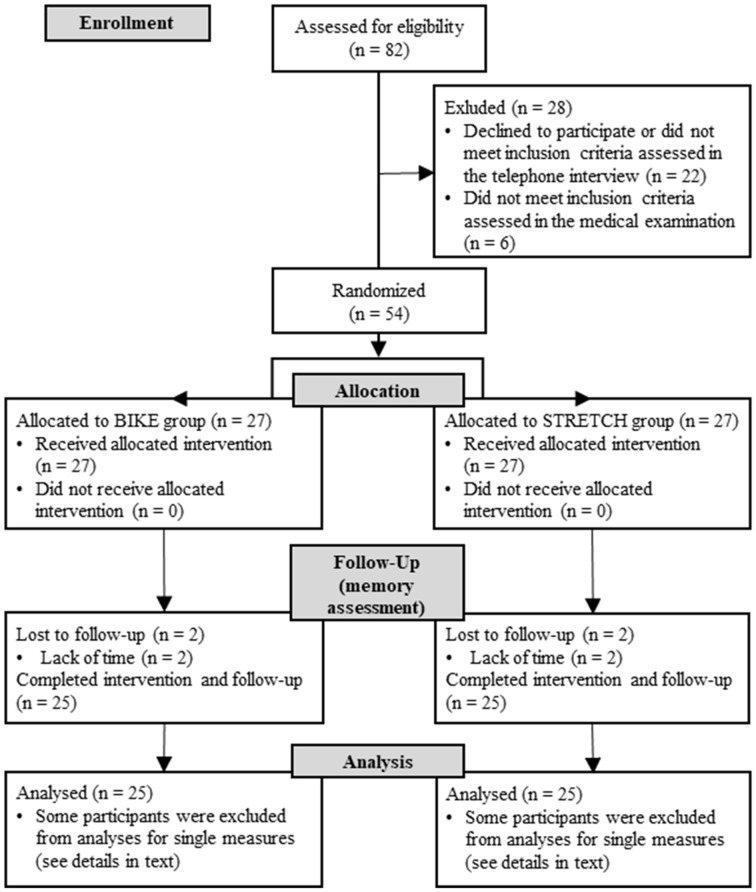
CONSORT flowchart of participants.

Eighty-two volunteers were recruited from the city of Hamburg (Germany) using flyers, public advertisements, and the online recruiting platform for psychological experiments at the University of Hamburg. Inclusion criteria comprised age of 18–40 years, an inactive lifestyle (on average ≤4 exercise sessions/month during the last 5 years), normal or corrected-to-normal vision, and normal hearing abilities. Exclusion criteria were chronic heart diseases, respiratory diseases, metabolic diseases, musculoskeletal disease, arthropathies, acute infections, chronic or acute neurological or psychiatric diseases, or treatment for neurological or psychiatric diseases in the past 3 years, regular alcohol consumption (>3 times/week), or the regular use of anti-inflammatory medication or medications known to affect the body’s immune response. Details of inclusion and exclusion of participants throughout the study are shown in Figure 1. The final sample consisted of 50 adults (37 females; age range = 20–40 years; *M* = 27.04, *SD* = 5.38).

Participants received monetary compensation for participation in all training sessions of the larger project. All procedures were carried out in accordance with the Helsinki Declaration guidelines (World Medical Association, 2013). The study was approved by the local ethical board of the Faculty of Psychology and Movement Science at the University of Hamburg. Written informed consent was obtained from all participants.

### Design

**Figure 2:**
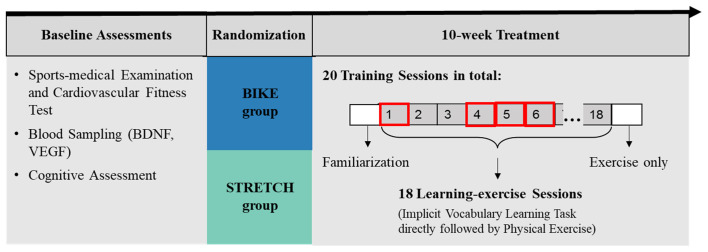
Graphical representation of the study design. This study addressed the effects of a single acute exercise session after learning on memory. Therefore, only retention of the vocabulary learned in the first learning-exercise session (learning-exercise session 1) was analyzed, which was measured 1–2 weeks after the initial acquisition (in learning-exercise sessions 4, 5, and 6).

Data presented in the present article were collected in the first 4 weeks of the longitudinal randomized training study. The larger training study took place over a period of 10 weeks and included multiple training sessions, each consisting of a learning task directly followed by physical exercise (Figure 2).

Before the first training session, each participant underwent baseline assessments including a sports-medical examination, a cardiorespiratory fitness test, and baseline blood sampling. Moreover, participants took part in a cognitive assessment, including measurement of verbal intelligence (*Mehrfachwahl-Wortschatz-Intelligenztest*; MWT-B; Helmstaedter et al., 2001) and filled in questionnaires on their physical activity (*Freiburger Fragebogen zur körperlichen Aktivität*; FFKA; Frey et al., 1999) and on depressive symptoms (*Allgemeine Depressionsskala*; ADS; Meyer and Hautzinger, 2001).

After baseline assessments, participants were stratified based on age (over and under 30 years) and then randomly assigned to either a cardiovascular training group (BIKE) or a stretching and toning group (STRETCH). While participants in the BIKE group participated in indoor cycling training, participants in the STRETCH group completed a light stretching and toning training. Over a period of 10 consecutive weeks, participants in both groups trained on average twice per week, resulting in a total of 20 exercise sessions for each participant. Eighteen of these training sessions were learning-exercise sessions in which participants exercised immediately following an implicit vocabulary learning task. During an initial familiarization session, participants were instructed in the use of the sports equipment and the training protocol. The present study focused only on the acute effects of physical exercise after learning in the first learning-exercise session. Therefore, only recognition of items from learning-exercise session 1, which was measured 1–2 weeks afterwards, were considered (Figure 2). The study took place between March 2018 and August 2019.

### Sports-Medical Examination and Cardiorespiratory Fitness Test

The sports-medical examination included a medical evaluation of the participants’ eligibility for the cardiorespiratory fitness test and participation in the physical exercise training. The examination included documentation of the medical history, a clinical examination, the recording of anthropometric data, urine examination, pulmonary function test, resting electrocardiogram (ECG), and a blood sampling (whole blood count, small blood count, liver enzymes, kidney enzymes, minerals, metabolic parameters, muscle enzymes, total protein, baseline measurement of neurotrophic factors). In addition, participants took part in a standardized stepwise incremental cycle ergometer test (Ergoline ER 900, Monark Ergometric 839E, Cosmed Ergoselect 4) to evaluate their peak oxygen uptake volume (VO_2_peak) and individual aerobic-anaerobic threshold. This test began with a warm-up period of 3 min at 50 watts, after which the workload was continuously increased in 50 watt steps every 3 min until subjects indicated complete exhaustion. Lactate measurements and blood pressure measurements were taken before, and every 3 min during the ergometry test as well as 1, 3, and 5 min after its completion. Heart rate and spirometer recordings were continuously measured.

WinLactat software (Mesics GmbH) was used to determine the individual aerobic-anaerobic threshold using lactate measurement, oxygen uptake, and anthropometric data. VO_2_peak was taken as a measure of cardiorespiratory fitness. For participants in the BIKE group, the target heart rate for the training was defined as 85% of the heart rate at the individual aerobic-anaerobic threshold, plus/minus five beats.

### Implicit Vocabulary Learning Task

For the vocabulary learning task, we adapted the experimental paradigm of Breitenstein and Knecht (2002). Participants repeatedly heard pseudowords while they simultaneously saw black-and-white images on a computer screen. Their task was to decide intuitively whether the presented pseudoword-picture pair was correct or incorrect. The ratio of correct to incorrect pseudoword-picture pairs increased over time. In this way, the more frequently shown pairs were learned to be *correct pairs*. This paradigm has been shown to elicit activity in the hippocampus (Breitenstein et al., 2005). In the study of Breitenstein and Knecht (2002), a total of 50 pseudoword-picture pairs were presented on five consecutive days. As the present study was part of a larger project, the learning task followed the same training principle as described by Breitenstein and Knecht (2002), but used a larger stimulus set to cover more learning-exercise sessions.

**Stimuli and Material**. Auditory stimuli were taken from a pool of 239 disyllabic German pseudowords, spoken by a female voice, and with a length of 600–1,000 ms (described in detail in Röder et al., 2003). A pilot study was conducted in which 24 participants (19 female; age range = 19–43 years; mean age = 23.8, *SD* = 5.16) rated these pseudowords in terms of their association with real words and pleasantness. Participants heard the pseudowords *via* over-ear headphones. It was their task to type in the perceived pseudoword as well as any associations with real words and to rate the pleasantness of the pseudoword on a scale from 1 to 5 (1 = very pleasant, 5 = very unpleasant). Pseudowords with scores at the extremes of these categories were discarded. Audacity^®^ recording and editing software version 2.1.3^1^ was used to either stretch or shorten the remaining pseudowords to a length of 800 ms. After the first author verified that length adjustments did not distort the sound, 150 disyllabic pseudowords, each 800 ms in length with little to no associations with real German words remained. Auditory stimuli were presented with over-ear headphones (AKG k518dj/Sennheiser HD65tv).

The Multilingual Picture (MultiPic) data bank of the Basque Center on Cognition, Brain, and Language was used to obtain the visual stimuli (BCBL; Duñabeitia et al., 2018). The data bank contains 750 drawings of common concrete concepts, which are standardized for visual complexity and name agreement in different languages. One-hundred and fifty black and white images were pre-selected by the first author. Thereafter, two research assistants ensured that the visual stimuli were not related to words that participants had associated with the pseudowords in the pilot study. Visual stimuli were presented with a size of 8 × 8 cm in the center of a computer screen. The distance between participants and the screen was approximately 80 cm.

**Figure 3:**
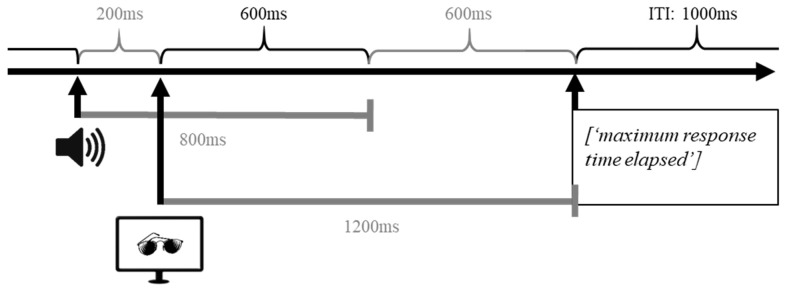
Graphical representation of the time course of a single trial in the implicit vocabulary learning task. In each trial, participants were first presented with an auditory stimulus (pseudoword); 200 ms after the onset of the auditory stimulus a visual stimulus (black-and-white picture) was presented until either participant made a response or the maximal response time was reached. If participants did not respond within the response interval of 1,200 ms, the message “maximum response time elapsed” was displayed on the screen. After 1,000 ms the next trial was initiated.

**Procedure**. The vocabulary learning task was programmed in Matlab with Psychtoolbox (R2016b, Mathworks Inc.; MATLAB, 2016). At the beginning of each trial, participants were presented with an auditory stimulus (pseudoword). After 200 ms, a visual stimulus (black-and-white picture) was presented until a response was made or the maximum response time of 1,200 ms was exceeded. In case a participant did not respond within the maximum response interval, the message “maximum response time elapsed” appeared on the computer screen and the next trial started. The intertrial interval (ITI) was set to 1,000 ms (Figure 3).

Participants’ task was to decide intuitively whether the presented pseudoword-picture pair was *correct* or *incorrect*. Responses were given by either pressing the left or the right button on a response device. Participants were instructed to use any two fingers of their dominant hand and to keep them constant across learning sessions. The assignment of *correct* and *incorrect* to the left and right buttons was counterbalanced across participants. Buttons were marked with the labels *correct* and *incorrect*, respectively. Feedback was given at the end of a learning session.

For each participant, a unique set of 150 correct pseudoword-picture pairs was randomly created when initiating the implicit vocabulary learning task for the first time (learning-exercise session 1). Within the learning session, participants were presented with 50 correct pseudoword-picture pairs. These *correct pairs* were mixed with randomly created *incorrect pairs*.

**Figure 4:**
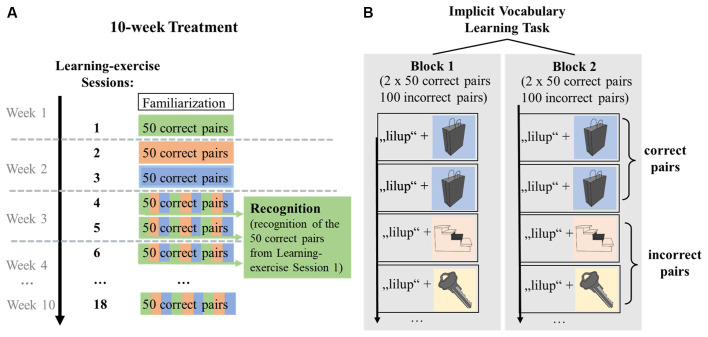
Graphical representation of the distribution of correct pseudoword-picture pairs over learning-exercise sessions, extraction of recognition performance and within-session learning. **(A)** Distribution of correct pseudoword-picture pairs across learning-exercise sessions. In learning-exercise sessions 1, participants encountered 50 correct pseudoword-picture pairs, which were randomly distributed across sessions 4, 5, and 6 (indicated by green color). To measure recognition of the vocabulary encoded in learning-exercise session 1, participants’ responses to the 50 correct pairs shown in session 1 were extracted from sessions 4, 5, and 6 (shown in green). Data of learning-exercise sessions 7–18 were not analyzed for the acute effects of exercise. **(B)** Within-session learning. A learning session was divided into two blocks. Within a block, each of the 50 correct pairs was shown twice (indicated by blue color), resulting in 100 correct pairs. By contrast, the 100 incorrect pairs consisted of 50 pseudowords presented twice but paired with different pictures, thus, resulting in 100 unique incorrect pairs per block. Each of the incorrect pairs was shown only once (indicated by yellow and orange color). This ratio of correct to incorrect pairs allowed learning within a session from block 1 to block 2. Stimuli were repeated in the second block, in the same order.

The first learning session comprised two identical blocks, with 100 correct and 100 incorrect pseudoword-picture pairs each. The 100 correct pairs within a block were composed of 50 correct pairs shown twice. By contrast, the 100 incorrect pairs consisted of 50 pseudowords presented twice but paired with different pictures, thus, resulting in 100 unique incorrect pairs. After a 5-min break, the block was repeated. The crucial difference between correct pairs and incorrect pairs was that exactly the same correct pairs were presented twice per block, but each incorrect pair was only encountered once. This presentation pattern led to a ratio of 2:1 for correct:incorrect pairs. The knowledge of correct and incorrect pseudoword-picture pairs was expected to build up from block 1 to block 2 (Figure 4B). At the end of the session, participants were given feedback by seeing the percentage of correct answers. This was calculated as the average percentage of correct answers over the two blocks.

Due to the general structure of the learning paradigm across 18 sessions in the larger study project, sessions 1, 2, and 3 each included a different set of 50 correct pseudoword-picture pairs, resulting in a total of 150 correct pseudoword-picture pairs. The 150 correct pairs were unique to each participant and remained the same for each participant across all sessions. These 150 correct pairs presented in sessions 1, 2, and 3 were then mixed with new incorrect pairs and randomly distributed across sessions 4, 5, and 6. The incorrect pairs changed in each session. This means that the 50 correct pairs in sessions 4, 5, and 6 were each composed of, on average, one-third of the correct pairs learned in sessions 1, 2, and 3, respectively (Figure 4A). Specifically, of the 50 correct pairs presented in session 1, on average, 16 pairs (min = 11, max = 20) were presented in session 4, 17 pairs (min = 12, max = 23) in session 5, and 17 pairs (min = 10, max = 23) in session 6. Across participants, session 4 took place on average 11.4 days (min = 6, max = 22), session 5 was on average 13.9 days (min = 10, max = 24), and session 6 was on average 16.9 days (min = 11, max = 25) after the first learning session.

D-prime (d’) values were calculated as a measure of the participants’ ability to distinguish between incorrect pairs and correct pairs. Thereby, correctly identified correct pairs were defined as *hits*; incorrect pairs falsely categorized as correct were defined as* false alarms*. D’ was calculated by subtracting the z-scores of the *false alarm rate* (= false alarms/number of incorrect trials) from the z-score of the* hit rate* (= hits/number of correct trials).

In this part of the project, the objective was to determine how many of the correct pairs that had been shown in learning-exercise session 1 were recognized as correct pairs during the second presentation, i.e., in sessions 4, 5, and 6. Therefore, responses to the 50 correct pairs that had been presented in learning-exercise session 1 were extracted from learning-exercise sessions 4, 5, and 6 for each participant (Figure 4A). The hit rate for the extracted 50 correct pairs was used to calculate recognition performance. False alarms were calculated by averaging the false alarm rate of sessions 4, 5, and 6; separately for blocks 1 and 2. Two measures of recognition performance were analyzed: the memory score extracted from the first blocks of sessions 4, 5, and 6 (d’ of block 1) and within-session learning (d’ from block 1 to block 2) for the extracted words from sessions 4, 5, and 6.

### Blood Sampling and Analysis of Neurotrophic Factors

At baseline and in one learning-exercise session, 7.5 ml blood was collected from the elbow vein. The baseline sample was taken during the sports-medical examination before the cardiovascular fitness test, and two further samples were collected in one of the first learning-exercise sessions (session 1, 2, 3, or 4), one sample directly before exercise and the second sample directly after exercise. Thus, each participant had two resting measurements, one at baseline and one directly before exercising, as well as one post-exercise measurement. In which of the learning-exercise sessions 1–4 the blood samples were taken was dependent on the availability of a qualified staff member. The number of participants assessed in learning-exercise sessions 1, 2, 3, and 4, respectively, did not differ between the BIKE and STRETCH group (Table 1).

**Table 1:** Frequency distribution of the learning-exercise sessions in which blood sampling was performed.

	Learning-exercise session 1	Learning-exercise session 2	Learning-exercise session 3	Learning-exercise session 4
BIKE	2	10	13	1
STRETCH	4	9	11	0

Baseline blood samples were centrifuged within 10 min after collection. For technical and organizational reasons, blood samples collected before exercise had to be stored at room temperature until the sampling after exercise had been performed. Therefore, the blood samples taken before exercise had a longer average time interval between collection and centrifugation, compared to the blood samples taken after exercise. Neurotrophic factor levels measured in blood serum could be influenced by clotting time with longer time intervals between blood collection and centrifugation increasing serum levels of BDNF and VEGF (Webb et al., 1998; Gejl et al., 2019). Comparing serum levels of BDNF and VEGF at baseline (during the medical examination) and immediately before exercising in the learning-exercise session 1, 2, 3, and 4, respectively, revealed a significantly larger mean level across participants for samples collected before exercising compared to baseline, although both were taken at rest. *Post hoc*, we ran a control experiment in four additional participants and varied the time intervals from blood sampling to centrifugation (0, 1.0 h, 1.5 h, 2.0 h, 3.0 h), which showed an increase in BDNF and VEGF levels during the 1st hour of clotting and a plateau for longer clotting times. Therefore, to assess the effect of acute exercise on neurotrophic factor levels, blood samples after exercise in sessions 1, 2, 3, and 4, respectively, were compared with the blood samples taken at baseline. Samples taken immediately before exercising in sessions 1, 2, 3, and 4, respectively, were not considered for analyses. On average, blood samples after exercise were taken 2 h later in the day than the baseline samples. However, this was the case in both groups, making it unlikely that circadian influences could account for group differences in exercise-induced changes in neurotrophic factors (Table 2). The blood collection procedure was the same for both groups. Thus, any group difference cannot be accounted for by how the blood samples were taken.

**Table 2:** Timing of blood sampling at baseline and after exercise as Mean and Standard Deviation in brackets.

	BIKE	STRETCH
Days between baseline and after exercise blood sampling (in days)	18.9 (6.88)	18.6 (8.91)
Time of baseline sampling (in hours)	12:58 (2:55)	12:53 (3:09)
Time of sampling after exercise (in hours)	14:45 (4:41)	14:10 (4:54)
Absolute time difference (after exercise - baseline; in hours)	4:43 (3:12)	3:50 (3:12)
Relative time difference (after exercise - baseline; in hours)^a^	−1:58 (5:25)	−1:10 (4:55)
	min = 7:30, max = −11:30	min = 7, max = −11

*^a^Negative values: blood sampling at baseline was conducted earlier in the day than blood sampling after exercise*.

All blood samples were centrifuged at room temperature for 10 min at 4,000 RPM. Three milliliters of isolated serum were filled in cryotubes and directly stored at −24°C until transported to the Bernhard Nocht Institute for Tropical Medicine in Hamburg (BNI) for storage at −80°C on nitrogen for later analysis of the serum concentration of BDNF and VEGF. BDNF concentrations were assessed with an ELISA kit (Human BDNF ELISA MAX Deluxe Set provided by BioLegend (Cat.No 446604, Lot 8276603). Quantification was performed with an ELISA Reader (Photometer). For analysis of VEGF, BioLegend’s LEGENDplex multiplex assay (Custom Human 9-plex Panel, BioLegend, USA) was used. 3 μl of each serum sample was diluted four-fold (1:4 dilution). Samples were placed on 96-well V-bottom Polypropylen plates provided by GreinerBio. For quantification, the ACCURI C6 FlowCytometer (Becton Dickenson) was used with a detection limit of 2.4 pg/ml.

The concentration of neurotrophic factors in pg/ml was used as a dependent variable. Due to errors in the laboratory analyses, data from one participant were missing for the baseline measurement (BIKE) and data from two participants were missing for both the baseline measurement and the measurement after exercise (one BIKE, one STRETCH).

## Physical Exercise

### Cardiovascular Exercise Training (BIKE)

Training sessions in the BIKE group included cardiovascular training on a cycle ergometer (Taurus Indoor Bike IC50) using video based indoor cycling instructions (CyberFitness GmbH^2^). Across training sessions, different videos were shown in a fixed order. These videos alternated between showing the instructor on an indoor bike and a first-person perspective of a cyclist riding through various landscapes. The videos included a short, low-impact warm-up, after which participants exercised for approximately 45–55 min at their target heart rate as determined by the cardiorespiratory fitness test. Participants were equipped with a fitness and activity tracker (Polar A300, Polar Electro Oy, Finland) which continuously recorded their heart rate. They were instructed to regularly check their heart rates on the activity tracker. The training ended with a cool-down and brief stretching.

### Stretching and Toning (STRETCH)

In the STRETCH group, training sessions were instructed *via* videos which were selected from an online fitness platform (fitnessRAUM.de GmbH^3^). Training sessions included a wide range of low-impact exercises. Among them were exercises to prevent back pain, instructions on how to sit and stand properly, light gymnastics such as sit-ups or low-impact push-ups, stretching, and relaxation exercises. Some videos invited participants to use equipment, such as a resistance band or a water bottle as a dumbbell. To match the training duration of the BIKE group, each training session in the STRETCH group contained several videos, which participants watched in a fixed order. Heart rate was continuously monitored, but participants were not asked to pay attention to their heart rate during the training.

## Statistical Analysis

Data were analyzed using the statistical software R (version 3.5.1, R Core Team, 2018). Independent samples t-tests were used to compare the BIKE and the STRETCH group at pre-assessment. The *lme4* package (Bates et al., 2015) was used to analyze performance in the vocabulary learning task and neurotrophic factor levels with linear mixed effect models (LMMs). Group was inserted as a factor in all models, with the STRETCH group serving as reference level. The fixed and random effects of each model are described in detail below. If not stated otherwise, all models included the covariates gender and age, which was centered at the mean. The package *parameters* (Lüdecke et al., 2020) were used for obtaining *p*-values and confidence intervals, using Wald-test approximation. *Post hoc* tests were performed with pairwise comparisons of estimated marginal means and the package *emmeans* (Lenth, 2019). Corrections for multiple comparisons were conducted following the Tukey method. The significance level was set to *p* < 0.05 for all analyses.

### Implicit Vocabulary Learning Task

Memory scores in the first block of the recognition sessions (d’ of block 1) were compared between the groups using an independent sample t-test. For analysis of within-session learning, an LMM was set up with d’ values of the recognition sessions as dependent variables. As fixed effects, the model included the interactions and main effects of group and block (block 1, block 2). *Block 1* served as the reference of the factor block. Random effects included individual intercepts for each participant as well as individual slopes for the effect of the block. This allowed participants an individual learning slope from block 1 to block 2. To account for different time intervals between learning-exercise session 1 and learning-exercise sessions 4–6, the mean time interval in days was inserted as a covariate. The main parameter of interest in this model was the interaction of group and block, to test whether groups differed in within-session learning.

### Neurotrophic Factors

A separate model was set up for each neurotrophic factor. Neurotrophic factor levels were included as the dependent variables. As fixed effects, models contained the interaction and main effects of time (baseline, after exercise) and group. *Baseline* served as the reference for the factor time. Random effects included individual intercepts for each participant. The main parameter of interest in this model was the interaction between group and time. This term described whether there were differences in the change of neurotrophic factors from baseline measurement to after exercise measurement between the two groups. Exclusion criteria for single data points were values more than three standard deviations above the mean. VEGF data of one participant of the STRETCH group were removed as outliers.

### Regression Analysis

Regression models were used to explore possible associations between changes in neurotrophic factor levels from baseline to after exercise and memory performance 1–2 weeks after learning (described in “Implicit Vocabulary Learning Task” section). Memory performance in the recognition sessions was indicated by two measures: memory score in the first blocks (d’ of block 1) and within-session learning, calculated as the difference in d’ from blocks 1 to blocks 2 (block 2 - block 1). Changes in BDNF and VEGF levels were calculated by subtracting the levels measured at baseline from those after exercise (after exercise - baseline). Change scores of neurotrophic factors were z-standardized before they were entered in the respective model. In total, four separate models were set up. In these models, the memory score in block 1 and within-session learning was predicted from the interaction of change in neurotrophic factor level and group. All models included the following covariates: age (centered at the mean), gender, the average number of days between session 1 and recognition, and the time difference (time of the day) between baseline and after exercise blood sampling. Pearson’s partial correlations (adjusted for covariates) were conducted to explore possible associations of memory with changes in neurotrophic factors in the single groups. For the change scores of BDNF and VEGF, participants were excluded if the change was more than three standard deviations above the mean. This was the case for VEGF of one participant in the BIKE group.

## Results

The BIKE and STRETCH groups did not differ in age, cardiorespiratory fitness (VO_2_peak), body mass index (BMI), and depression score at baseline (Table 3).

**Table 3:** Group characteristics at baseline as Mean with Standard Deviation in brackets.

	BIKE	STRETCH	p-value^a^ [95% CI]
n	25	25	
Male/Female	6/19	6/19	
Age	27.4 (5.82)	26.7 (5.03)	0.679, [–3.73, 2.45]
Verbal intelligence^b^	99.2 (10.10)	107.1 (12.55)	**0.030**, [0.82, 14.92]
VO_2_peak	33.7 (5.89)	34.3 (6.14)	0.732, [–2.84, 4.01]
BMI	24.2 (4.03)	23.5 (3.73)	0.523, [–2.92, 1.50]
Depression Score	12.9 (6.83)	12.4 (5.27)	0.747, [–4.03, 2.92]
D’ of Block 1 in the first learning-exercise session	0.11 (0.31)	0.19 (0.21)	0.225, [–0.24, 0.06]
Within-session learning in the first learning-exercise session (Block 2 - Block 1)	0.15 (0.31)	0.17 (0.34)	0.834, [–0.20, 0.17]

*Note. Bold print indicates significance at *p* < 0.05. ^a^Independent *t*-test; ^b^Measured with the MWT-B. MWT-B data of seven participants who were non-native German speakers (4 BIKE group, 3 STRETCH group) were excluded from the analysis*.

In the first learning-exercise session, the BIKE group trained with a significantly higher heart rate (*M* = 143 beats/min, *SD* = 13.83) compared to the STRETCH group (*M* = 92 beats/min, *SD* = 8.55); *t*_(40.26)_ = 15.42, 95% CI: [43.8, 57.0], *p* < 0.001.

Comparing d’ in block 1 and within-session learning in the first learning-exercise session revealed no significant difference between the BIKE and the STRETCH group (Table 3; no significant group × block interaction *(ß* = −0.02, 95% CI [−0.21, 0.17], *p* = 0.838).

### Implicit Vocabulary Learning Task

The comparison of d’ in block 1 of the recognition sessions revealed no significant difference between the groups; *t*_(46.80)_ = −0.30, 95% CI [–0.25, 0.19], *p* = 0.762.

**Figure 5:**
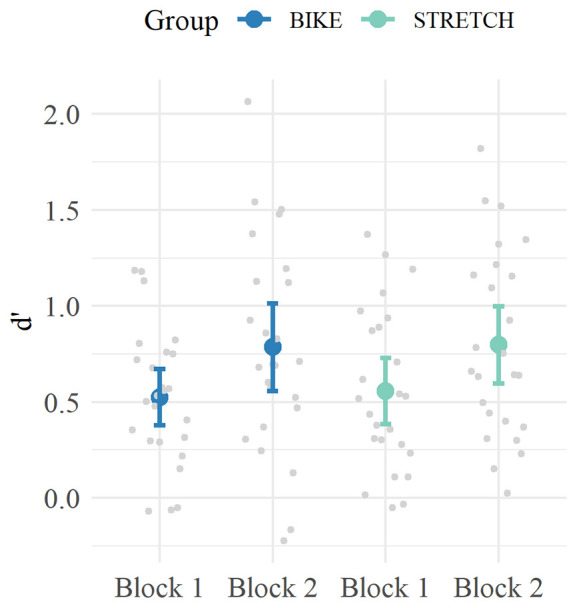
Mean d‘ values for Block 1 and Block 2 of the recognition sessions. For each participant, the data of the correct pairs encountered in learning-exercise session 1 were extracted from learning-exercise sessions 4–6 and used to calculate d’ values. Means are depicted in blue for the BIKE group and in green for the STRETCH group. Error bars depict 95% confidence intervals. Data of single participants are depicted in gray.

Analysis of d’ values from block 1 to block 2 revealed a significant main effect of block *(ß* = 0.24, 95% CI [0.14, 0.34], *p* < 0.001) but no significant group × block interaction *(ß* = 0.02, 95% CI [–0.12, 0.16], *p* = 0.774). Hence, participants of both groups increased their performance from block 1 to block 2, but there was no difference in within-session learning between the groups. In sum, when encountering the correct pseudoword-picture pairs learned in session 1 a second time, there was no difference in memory performance between the BIKE and the STRETCH group (Figure 5).

### Neurotrophic Factors

Analysis of BDNF levels from baseline to after exercise showed a significant main effect of time (*ß* = 591, 95% CI, [154, 1,028], standardized* ß* = 0.32, *p* = 0.008), and a significant interaction of time × group (*ß* = 715, 95% CI [95, 1,336], standardized* ß* = 0.34, *p* = 0.037). *Post hoc* tests indicated that both groups had significantly higher BDNF levels after exercise compared to baseline. However, this increase was significantly larger in the BIKE (after exercise—baseline: *ß* = 1,307, 95% CI [853, 1,760], *p* < 0.001) compared to the STRETCH group (*ß* = 591, 95% CI [143, 1,039], *p* = 0.011).

**Figure 6:**
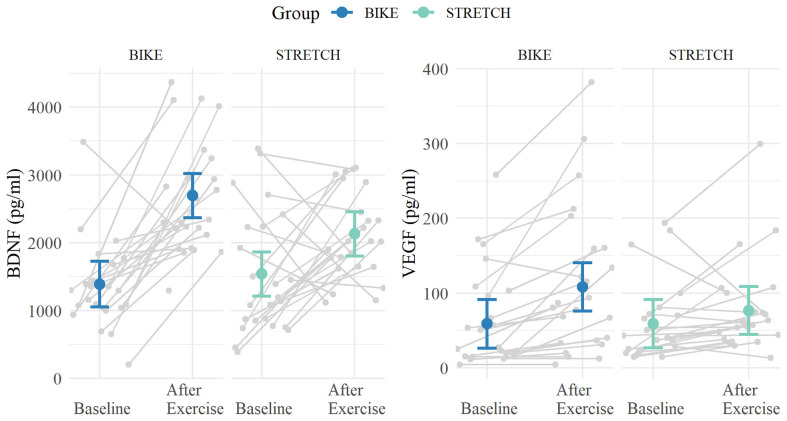
Means of BDNF and VEGF levels at baseline and directly after one bout of exercise. Blood was collected at rest in the sports medical examination (baseline) and directly after one bout of exercise in one of the first learning-exercise sessions 1–4. Means are depicted in blue for the BIKE group and in green for the STRETCH group. Error bars depict 95% confidence intervals. Data of single participants are depicted in gray.

Results for VEGF from baseline to after exercise yielded a significant interaction of time × group (*ß* = 31.6, 95% CI, [1.68, 61.49], standardized* ß* = 0.19, *p* = 0.038). *Post hoc* tests indicated the BIKE group’s VEGF levels significantly increased after exercise (after exercise—baseline: *ß* = 49.5, 95% CI [28.0, 71.0], *p* < 0.001) while showing no significant change for the STRETCH group (after exercise—baseline: *ß* = 17.8, 95% CI [−4.0, 39.5], *p* = 0.107). Both the changes in BDNF and VEGF level are depicted in Figure 6.

To test whether the time of day might have influenced the effects, we ran additional models including the time of blood sampling as covariate (as numeric from 0:00 h). Including the time of blood sampling into the model did not change the pattern of results and the group × time interaction of both models remained significant (BDNF time × group: *p* = 0.027, VEGF time × group: *p* = 0.037).

### Associations of Changes in Neurotrophic Factor Levels With Memory

We tested whether the exercise-induced increase in neurotrophic factors correlated with the memory score in block 1 and with within-session learning in the recognition sessions.

**Figure 7:**
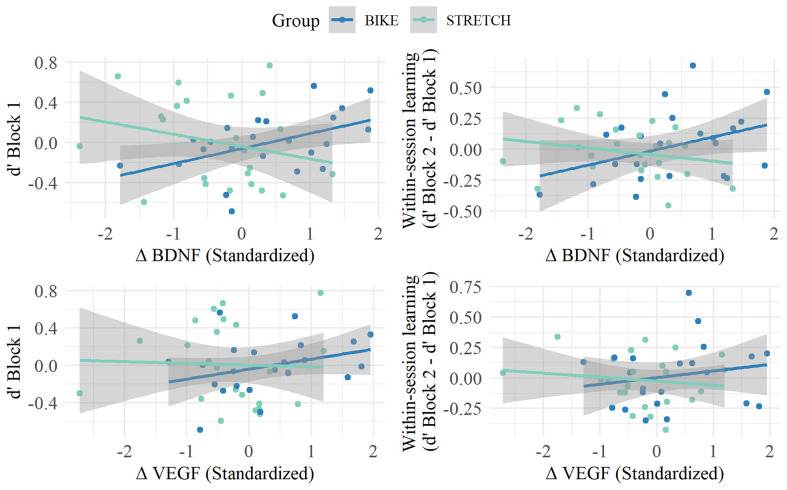
Associations between memory score in Block 1 (left) and within-session learning (right) in the recognition sessions and the change in BDNF (top) and VEGF (lower) levels from baseline to after exercise. Partial residual plots of the relationship between memory in the recognition sessions and the standardized change in neurotrophic factor levels (BDNF, VEGF) from baseline to after a single bout of exercise (after exercise - baseline); r adjusted for age, gender, the average number of days between learning-exercise session 1 and recognition, and the time difference between baseline and after exercise blood sampling. Regression lines are blue for the BIKE group and green for the STRETCH group. Circles in the respective colors represent single participants. Gray shades indicate 95% confidence bands.

For the memory score in block 1, models revealed a marginal significant interaction between the change in BDNF and group (*ß* = 0.26, standardized *ß* = 0.44, 95% CI [–0.001, 0.512], *p* = 0.051). Partial correlations, separately for each group, indicated a marginal significant correlation between BDNF increase and memory score in the first block for the BIKE group (*r*_(23)_ = 0.41, *p* = 0.082), but not in the STRETCH group (*r*_(24)_ = −0.21, *p* = 0.364). For within-session learning, models indicated a significant interaction between the change in BDNF and group (*ß* = 0.18, standardized *ß* = 0.46, 95% CI [0.02, 0.33]; *p* = 0.035). Partial correlations revealed a marginally significant positive association between the change in BDNF and within-session learning in the BIKE group (*r*_(23)_ = 0.40, *p* = 0.086), but not in the STRETCH group (*r*_(23)_ = −0.27, *p* = 0.253). Partial correlations are depicted in Figure 7. We tested whether these correlations may be confounded by possible relationships between baseline BDNF and memory. Results showed no significant correlation between baseline BDNF levels and the memory score extracted from the first blocks of sessions 4, 5, and 6 (d’ of block 1; *r*_(47)_ = −0.06, *p* = 0.725) and within-session learning (d’ from block 1 to block 2) for the extracted words from sessions 4, 5, and 6 (*r*_(47)_ = −0.12, *p* = 0.429).

For both memory measures, there were no significant interactions between group and change in VEGF levels from baseline to after exercise (all *p* > 0.221). Collapsing data of both groups did not show an association between VEGF change and memory measures (all *p* > 0.550).

## Discussion

The aim of this study was to test whether a single bout of cardiovascular exercise carried out in the early stages of memory consolidation improves memory as assessed with an artificial vocabulary learning task. In addition, the effects of physical exercise on serum levels of BDNF and VEGF were determined in order to explore whether they mediate exercise-induced memory changes. Results did not indicate a beneficial effect of cycling on memory measured 1–2 weeks after initial acquisition compared to stretching and toning. Analyses of serum neurotrophic factor levels revealed significantly larger BDNF and VEGF increases after physical exercise in the cycling group compared to the stretching and toning group. Exercise-induced changes in BDNF levels tended to positively correlate with memory measures in the BIKE group, but not in the STRETCH group.

It has been hypothesized that physical exercise might enhance memory, possibly through the acute exercise-induced release of neuromodulatory factors, such as dopamine, norepinephrine, cortisol, and BDNF, which are known to be involved in memory consolidation (McGaugh, 2000; Siette et al., 2014; van Dongen et al., 2016; Miranda et al., 2019). In the present study, participants exercised immediately after encoding a new vocabulary. Thus, encoding conditions were held constant across groups, but the activity of the groups differed in the early stages of memory consolidation. Yet, the results of the present study did not provide evidence that cycling directly after the encoding phase was beneficial for memory consolidation, compared to stretching and toning. The timing of exercise relative to memory encoding has been discussed as a crucial factor modulating the benefits of acute exercise on memory processes (Roig et al., 2016). A meta-analysis reported larger effect sizes for memory improvements when exercise was implemented before than after learning (Roig et al., 2013). The larger effect sizes might be due to the combined effect of exercise on encoding and consolidation processes because the physiological adaptations induced by a bout of cardiovascular exercise performed before encoding are likely to persist into the early stages of memory consolidation. Roig et al. (2016) suggested that a close temporal coupling of exercise with memory processes is the key factor for memory improvements to occur. This is supported by studies that systematically varied the timing of exercise relative to a memory task. Results showed beneficial effects of an acute bout of cardiovascular exercise on visuo-motor memory regardless of whether exercise was performed immediately before or after motor learning, but not when exercise and learning were separated by 1 h longer time intervals (Statton et al., 2015; Thomas et al., 2016a). van Dongen et al. (2016) did not report beneficial effects of cardiovascular exercise on memory for picture-location associations and hippocampal pattern separation when exercise was performed immediately after learning. However, exercise improved memory when performed 4 h after learning, thus, in the late stages of memory consolidation (van Dongen et al., 2016). The authors discussed that levels of neurotrophins supporting synaptic plasticity might be naturally lower several hours after learning and thus, exercise upregulating the release of these factors might have a larger impact on memory outcomes during late consolidation stages. However, these explanations are still speculative and a possible more pronounced effect of delayed exercise relative to encoding on memory consolidation needs replication in further studies.

Moreover, studies differed with regard to the memory tasks used and thus, the addressed underlying neuronal networks. In the present study, we assessed the retention of items encoded in an implicit paired association task. fMRI data suggested that successful learning in this task is associated with hippocampal activity and increased functional coupling between the left hippocampus and cortical association areas as the left fusiform gyrus and the left inferior parietal lobe (Breitenstein et al., 2005). Results of previous acute exercise studies assessing memory in associative learning tasks have yielded inconsistent results with some studies showing better memory for associations encoded prior to exercise (van Dongen et al., 2016; Bosch et al., 2017a), while others did not find better memory for associations learned before exercise (McNerney and Radvansky, 2015; Hötting et al., 2016). Findings seemed to be more consistent for motor learning tasks. For instance, several studies reported positive effects of a single cardiovascular exercise session after practicing visuo-motor tracking tasks (Roig et al., 2012; Thomas et al., 2016b; Dal Maso et al., 2018). Learning in visuo-motor tasks has been shown to activate the basal ganglia, cerebellum, and motor cortices (Doyon et al., 2009) and have been found to be spared after hippocampal lesions (Corkin, 1968). Therefore, one could speculate that in humans, the brain structures responsible for motor learning are in particular sensitive to the beneficial effects of acute cardiovascular exercise. Yet, this is contradictory to results in rodents reporting very reliable exercise-induced functional and structural changes in the hippocampus after exercise (reviewed in Cotman et al., 2007; van Praag, 2008). Moreover, fMRT results in humans showed task-dependent modulations of hippocampal activity after acute exercise (Bosch et al., 2020). Future studies contrasting hippocampus-dependent learning tasks and hippocampus-independent learning tasks in humans might shed light on the question of whether there is a task-dependent effect of physical exercise in phases of early memory consolidation (McNerney and Radvansky, 2015).

In addition, studies analyzing the effects of acute cardiovascular exercise on memory differed in the intensity of the physical exercise and the fitness status of participants (Roig et al., 2013). In the present study, the BIKE group received cardiovascular training of moderate intensity. The training heart rates of participants in the BIKE group were adjusted to the individual aerobic-anaerobic threshold. Thus, the relative training intensity was similar for all participants and we made sure that participants trained within the aerobic range. Moreover, the heart rate was measured in the STRETCH group as well, confirming a significant mean difference of 44 beats/min between groups. Some recent studies have suggested that higher physical exercise intensities more reliably improve memory performance in acute exercise designs compared to low or moderate intensity exercise (Winter et al., 2007; Etnier et al., 2016; Thomas et al., 2016b). However, the effect of training intensity is additionally dependent on individuals’ baseline fitness and age.

It has been shown that more fit and regularly active participants showed stronger increases in cognitive functions after acute cardiovascular exercise compared to less fit and untrained peers (Chang et al., 2012; Hopkins et al., 2012). Participants in the present study were sedentary and had relatively low cardiovascular fitness levels (average peak oxygen uptake volume, VO_2_peak, of 34 ml/kg/min) compared to normative samples in that age range (Laukkanen and Held, 1999). Most other studies reporting positive effects of acute cardiovascular exercise on memory included participants with higher fitness levels of above a VO_2_peak of 40 ml/kg/min (Winter et al., 2007; Etnier et al., 2016; Thomas et al., 2016b; Bosch et al., 2017b). It remains a task of future research to further elucidate the extent to which variables such as prior physical activity, intensity of the physical exercise, or the interaction of both factors influence the effect of acute cardiovascular exercise on memory.

Due to the general study design of the larger project, memory for the pseudoword-picture pairs of the implicit vocabulary learning task was assessed 1–2 weeks after initial encoding and was spread over three separate sessions that took place on different days. Moreover, participants were exposed to further pseudoword-picture pairs between initial encoding and memory assessment, possibly causing interference effects. It is, therefore, possible that the study design may have masked potential effects on the behavioral level.

Exercise-induced changes in neurotrophic factor levels have been suggested to at least partly mediate the positive effects of cardiovascular exercise on memory (Cotman et al., 2007; El-Sayes et al., 2019). Consistent with the literature, BDNF levels increased significantly more after acute cardiovascular exercise compared with non-cardiovascular exercise (Winter et al., 2007; Hötting et al., 2016; Tsai et al., 2018). So far, only a few studies have investigated the response of VEGF levels to acute cardiovascular exercise and reported mixed findings (Landers-Ramos et al., 2014; Skriver et al., 2014; Tsai et al., 2018; Kujach et al., 2020). The present results indicated an increase of VEGF levels after cycling, but not after stretching and toning. Thus, a bout of moderate intensity cardiovascular exercise seems to increase both BDNF and VEGF levels in young, untrained adults.

Regarding the relationship between exercise-induced changes in BDNF levels and memory, results yielded significant differences between the BIKE and STRETCH groups. Follow-up analyses of the present study showed marginally significant associations between a larger increase in BNDF and better performance in measures of memory (memory performance in block 1 and within-session learning in the recognition sessions) only in the BIKE group. Correlation coefficients were of moderate strength. The sample size calculation for the present study was based on the main hypothesis of the larger project predicting a group difference in memory improvements across multiple learning sessions. Thus, the relatively small sample size in the BIKE group was not sufficient to test for significant correlations in such a range and needs replication in larger samples. Nonetheless, results of the present study add to the literature showing a positive relationship between the increase in BDNF and memory performance, suggesting that BDNF may be related to memory improvement after exercise (Schmidt-Kassow et al., 2014; Skriver et al., 2014; but see Etnier et al., 2016; Bosch et al., 2017a,b).

Although BDNF correlated with memory in the BIKE group, there was no difference in memory parameters between the BIKE and the STRETCH group. It has been assumed that higher levels of neuromodulatory substances (BDNF, VEGF) after encoding might facilitate memory consolidation (Miranda et al., 2019; van Dongen et al., 2016). BDNF levels in response to cardiovascular exercise have been shown to increase in an intensity-dependent manner, with higher intensities inducing larger increases (Ferris et al., 2007; Winter et al., 2007; Etnier et al., 2016; Hötting et al., 2016). Moreover, exercising with higher intensity than in the present study is known to increase levels of further neuromodulators, such as noradrenalin, adrenalin, and lactate (Winter et al., 2007; Skriver et al., 2014; Basso and Suzuki, 2017). It could, thus, be speculated that the cycling intensity in the present study did not sufficiently enhance BDNF levels and did not induce the secretion of neurochemical substances which would have been required for behavioral benefits after acute cardiovascular exercise (but see Bosch et al., 2017b for contrasting evidence).

In line with earlier research, the increase in VEGF did not correlate with memory and no group differences were found for the relationship between acute exercise-induced changes in VEGF levels and memory (Skriver et al., 2014). However, literature relating to exercise-induced VEGF changes and memory in both chronic and acute study designs is limited, and results are equivocal (Skriver et al., 2014; Woost et al., 2018). In observational studies, higher VEGF levels have been positively associated with larger hippocampal volume, less hippocampal atrophy, and less cognitive decline over time (Hohman et al., 2015) as well as with a decreased risk for Alzheimer’s disease (Mateo et al., 2007), indicating that VEGF might be beneficial for memory-related processes.

Results from rodent studies have supported the involvement of BNDF and VEGF in exercise-induced memory improvements and their associated structural changes in the brain, such as synaptogenesis, neurogenesis, and angiogenesis (Fabel et al., 2003; Vaynman et al., 2004; Cotman et al., 2007; Uysal et al., 2015; El-Sayes et al., 2019). While VEGF has mainly been related to neurogenesis and the growth and protection of the vasculature (Greenberg and Jin, 2005; El-Sayes et al., 2019), BDNF, in particular, has received a lot of attention due to its role in long-term potentiation, that is synaptic plasticity essential for memory consolidation (Miranda et al., 2019). Methodological differences between rodent and human research may partially explain inconsistent results found with respect to associations between neurotrophic factor levels and memory in humans (Schmidt-Kassow et al., 2014; Skriver et al., 2014). In contrast to rodents, invasive procedures to manipulate or measure levels of neurotrophic factors in the brain cannot be applied in humans. Therefore, they are typically measured peripherally in serum or plasma. It is unclear how these peripherally measured neurotrophic factors are related to the levels in the brain (Pan et al., 1998; Lanz et al., 2012; Rich et al., 2017). Moreover, local increases, for example in the hippocampus, cannot be determined by peripheral measures. Therefore, systemic measurements in humans might not reliably capture changes in neurotrophic factors and their relationship to memory, as they may increase and act particularly at the local level in the brain.

Among the limitations of the present study is the blood sampling procedure. Serum BDNF levels were increased after lightly exercising in the STRETCH group (average heart rate of 94 beats/min). Previous studies did not report BDNF increases after exercising at comparable intensity (Schmidt-Kassow et al., 2014; Hötting et al., 2016). For organizational and technical reasons, blood samples collected after exercise were stored at room temperature until they were transported to the centrifuge. This storage possibly caused the release of BDNF from platelets and may be responsible for the unexpected increase of BDNF levels in the STRETCH group (Webb et al., 1998; Gejl et al., 2019). Thus, the absolute values of neurotrophic factors in the present study should be interpreted with caution, in particular when comparing absolute serum values to those reported in previous studies. Nevertheless, given that the sampling procedure and storage time were the same for both groups, it is possible to unequivocally interpret group differences in BDNF change. Additionally, taking blood samples on different days and at different times might have introduced additional variance in the levels of neurotrophic factors due to time- and day-dependent fluctuations (Hetland et al., 2008; Piccinni et al., 2008). However, the time difference and number of days between baseline and post-exercise blood sampling were similar between the BIKE and STRETCH groups, rendering it highly unlikely that circadian fluctuations accounted for group differences.

The present data show no beneficial effect of a single cardiovascular exercise session compared with a single stretching and toning session on early stages of memory consolidation in young adults. However, acute cardiovascular exercise increased both BDNF and VEGF levels in comparison to non-cardiovascular exercise. Moreover, positive correlations between changes in BDNF and memory measures were compatible with the idea and previous findings (Skriver et al., 2014; Bosch et al., 2017b) that BDNF contributed to memory enhancement after acute cardiovascular exercise.

## Data Availability Statement

The raw data analyzed in this study are available on request from the corresponding author. The data are not publicly available due to privacy concerns.

## Ethics Statement

The studies involving human participants were reviewed and approved by the Local Ethics Committee of the Faculty of Psychology and Human Movement Science, University of Hamburg. The participants provided their written informed consent to participate in this study.

## Author Contributions

LK contributed to the study concept and design, recruitment of participants, implementation, data management, data analysis, interpretation of results, and drafted the manuscript. A-MK contributed to the sports medical data collection, data management, data analysis, and to the critical review of the manuscript. K-MB and RR contributed to the study concept and critical review of the manuscript. TJ contributed to the study concept, analysis of BDNF and VEGF data and interpretation, and critical review of the manuscript. BR contributed to the study concept and design, interpretation of results, critical review of the manuscript, and secured funding for the study. KH contributed to the study concept and design, data analysis, interpretation of results, and critical review of the manuscript. All authors contributed to the article and approved the submitted version.

## Conflict of Interest

Cyberfitness.tv and FitnessRaum.de provided the training videos used in the physical exercise sessions free of charge. The companies were not involved in the study design, collection, analysis, interpretation of data, the writing of this article or the decision to submit it for publication.

## Publisher’s Note

All claims expressed in this article are solely those of the authors and do not necessarily represent those of their affiliated organizations, or those of the publisher, the editors and the reviewers. Any product that may be evaluated in this article, or claim that may be made by its manufacturer, is not guaranteed or endorsed by the publisher.

## Footnotes

^1^Audacity^®^ software is copyright © 1999-2020 Audacity Team. The name Audacity^®^ is a registered trademark of Dominic Mazzoni.

^2^ https://www.cyberconcept.de/cybercycling/

^3^ https://www.fitnessraum.de/

## References

[B1] Anderson L. A. McConnell S. R. (2007). Cognitive health: an emerging public health issue. Alzheimers Dement. 3, S70–S73. 10.1016/j.jalz.2007.01.018 19595979

[B3] Basso J. C. Shang A. Elman M. Karmouta R. Suzuki W. A. (2015). Acute exercise improves prefrontal cortex but not hippocampal function in healthy adults. J. Int. Neuropsychol. Soc. 21, 791–801. 10.1017/S135561771500106X 26581791

[B2] Basso J. C. Suzuki W. A. (2017). The effects of acute exercise on mood, cognition, neurophysiology and neurochemical pathways: a review. Brain Plast. 2, 127–152. 10.3233/BPL-160040 29765853 PMC5928534

[B100] Bates D. Mächler M. Bolker B. Walker S. (2015). Fitting linear mixed-effects models using lme4. J. Stat. Softw. 67, 1–48. 10.18637/jss.v067.i01

[B4] Bekinschtein P. Cammarota M. Medina J. H. (2014). BDNF and memory processing. Neuropharmacology 76, 677–683. 10.1016/j.neuropharm.2013.04.024 23688925

[B5] Bosch B. M. Bringard A. Ferretti G. Schwartz S. Iglói K. (2017a). Effect of cerebral vasomotion during physical exercise on associative memory, a near-infrared spectroscopy study. Neurophotonics 4:041404. 10.1117/1.NPh.4.4.041404 28785600 PMC5526475

[B6] Bosch B. M. Bringard A. Logrieco M. G. Lauer E. Imobersteg N. Thomas A.. (2017b). Acute physical exercise improves memory consolidation in humans *via* BDNF and endocannabinoid signaling. BioRxiv [Preprint]. 10.1101/211227

[B7] Bosch B. M. Bringard A. Logrieco M. G. Lauer E. Imobersteg N. Thomas A.. (2020). Effect of acute physical exercise on motor sequence memory. Sci. Rep. 10:15322. 10.1038/s41598-020-72108-1 32948800 PMC7501852

[B9] Breitenstein C. Jansen A. Deppe M. Foerster A. F. Sommer J. Wolbers T.. (2005). Hippocampus activity differentiates good from poor learners of a novel lexicon. Neuroimage 25, 958–968. 10.1016/j.neuroimage.2004.12.019 15808996

[B8] Breitenstein C. Knecht S. (2002). Development and validation of a language learning model for behavioral and functional-imaging studies. J. Neurosci. Methods 114, 173–179. 10.1016/s0165-0270(01)00525-8 11856568

[B10] Cetinkaya C. Sisman A. R. Kiray M. Camsari U. M. Gencoglu C. Baykara B.. (2013). Positive effects of aerobic exercise on learning and memory functioning, which correlate with hippocampal IGF-1 increase in adolescent rats. Neurosci. Lett. 549, 177–181. 10.1016/j.neulet.2013.06.012 23792196

[B11] Chang Y. K. Labban J. D. Gapin J. I. Etnier J. L. (2012). The effects of acute exercise on cognitive performance: a meta-analysis. Brain Res. 1453, 87–101. 10.1016/j.brainres.2012.02.068 22480735

[B12] Chapman S. B. Aslan S. Spence J. S. DeFina L. F. Keebler M. W. Didehbani N.. (2013). Shorter term aerobic exercise improves brain, cognition and cardiovascular fitness in aging. Front. Aging Neurosci. 5:75. 10.3389/fnagi.2013.00075 24282403 PMC3825180

[B13] Coles K. Tomporowski P. D. (2008). Effects of acute exercise on executive processing, short-term and long-term memory. J. Sports Sci. 26, 333–344. 10.1080/02640410701591417 18074301

[B14] Corkin S. (1968). Acquisition of motor skill after bilateral medial temporal-lobe excision. Neuropsychologia 6, 255–265. 10.1016/0028-3932(68)90024-9

[B15] Cotman C. W. Berchtold N. C. Christie L.-A. (2007). Exercise builds brain health: key roles of growth factor cascades and inflammation. Trends Neurosci. 30, 464–472. 10.1016/j.tins.2007.06.011 17765329

[B16] Dal Maso F. Desormeau B. Boudrias M. H. Roig M. (2018). Acute cardiovascular exercise promotes functional changes in cortico-motor networks during the early stages of motor memory consolidation. NeuroImage 174, 380–392. 10.1016/j.neuroimage.2018.03.029 29555428

[B17] Ding Q. Vaynman S. Akhavan M. Ying Z. Gomez-Pinilla F. (2006). Insulin-like growth factor I interfaces with brain-derived neurotrophic factor-mediated synaptic plasticity to modulate aspects of exercise-induced cognitive function. Neuroscience 140, 823–833. 10.1016/j.neuroscience.2006.02.084 16650607

[B18] Doyon J. Bellec P. Amsel R. Penhune V. Monchi O. Carrier J.. (2009). Contributions of the basal ganglia and functionally related brain structures to motor learning. Behav. Brain Res. 199, 61–75. 10.1016/j.bbr.2008.11.012 19061920

[B19] Duñabeitia J. A. Crepaldi D. Meyer A. S. New B. Pliatsikas C. Smolka E.. (2018). MultiPic: a standardized set of 750 drawings with norms for six European languages. Q. J. Exp. Psychol. (Hove) 71, 808–816. 10.1080/17470218.2017.1310261 28326995

[B20] El-Sayes J. Harasym D. Turco C. V. Locke M. B. Nelson A. J. (2019). Exercise-induced neuroplasticity: a mechanistic model and prospects for promoting plasticity. Neuroscientist 25, 65–85. 10.1177/1073858418771538 29683026

[B21] Erickson K. I. Voss M. W. Prakash R. Basak C. Szabo A. Chaddock L.. (2011). Exercise training increases size of hippocampus and improves memory. Proc. Natl. Acad. Sci. U S A 108, 3017–3022. 10.1073/pnas.1015950108 21282661 PMC3041121

[B22] Etnier J. L. Wideman L. Labban J. D. Piepmeier A. T. Pendleton D. M. Dvorak K. K.. (2016). The effects of acute exercise on memory and brain-derived neurotrophic factor (BDNF). J. Sport Exerc. Psychol. 38, 331–340. 10.1123/jsep.2015-0335 27385735

[B23] Fabel K. Fabel K. Tam B. Kaufer D. Baiker A. Simmons N.. (2003). VEGF is necessary for exercise-induced neurogenesis. Eur. J. Neurosci. 18, 2803–2812. 10.1111/j.1460-9568.2003.03041.x 14656329

[B24] Fernandez A. M. Torres-Alemán I. (2012). The many faces of insulin-like peptide signalling in the brain. Nat. Rev. Neurosci. 13, 225–239. 10.1038/nrn3209 22430016

[B25] Ferris L. T. Williams J. S. Shen C.-L. (2007). The effect of acute exercise on serum brain-derived neurotrophic factor levels and cognitive function. Med. Sci. Sports Exerc. 39, 728–734. 10.1249/mss.0b013e31802f04c7 17414812

[B102] Frey I. Berg A. Grathwohl D. Keul J. (1999). Freiburger Fragebogen zur körperlichen Aktivität. Sozial- Und Palliativmedizin 44, 55–64. 10.1007/BF01667127 10407953

[B26] Gejl A. K. Enevold C. Bugge A. Andersen M. S. Nielsen C. H. Andersen L. B. (2019). Associations between serum and plasma brain-derived neurotrophic factor and influence of storage time and centrifugation strategy. Sci. Rep. 9:9655. 10.1038/s41598-019-45976-5 31273250 PMC6609657

[B27] Gourgouvelis J. Yielder P. Clarke S. T. Behbahani H. Murphy B. (2018). You can’t fix what isn’t broken: Eight weeks of exercise do not substantially change cognitive function and biochemical markers in young and healthy adults. PeerJ 2018:e4675. 10.7717/peerj.4675 29686948 PMC5911384

[B28] Greenberg D. A. Jin K. (2005). From angiogenesis to neuropathology. Nature 438, 954–959. 10.1038/nature04481 16355213

[B29] Griffin É. W. Mullally S. Foley C. Warmington S. A. O’Mara S. M. Kelly Á. M. (2011). Aerobic exercise improves hippocampal function and increases BDNF in the serum of young adult males. Physiol. Behav. 104, 934–941. 10.1016/j.physbeh.2011.06.005 21722657

[B31] Hedden T. Gabrieli J. D. E. (2004). Insights into the ageing mind: a view from cognitive neuroscience. Nat. Rev. Neurosci. 5, 87–96. 10.1038/nrn1323 14735112

[B101] Helmstaedter C. Lendt M. Lux S. (2001). Verbaler Lern- und Merkfähigkeitstest. Göttingen: Hogrefe.

[B32] Hetland M. L. Christensen I. J. Lottenburger T. Johansen J. S. Svendsen M. N. Hørslev-Petersen K.. (2008). Circulating VEGF as a biological marker in patients with rheumatoid arthritis? Preanalytical and biological variability in healthy persons and in patients. Dis. Markers 24, 1–10. 10.1155/2008/707864 18057530 PMC3850601

[B33] Hohman T. J. Bell S. P. Jefferson A. L. (2015). The role of vascular endothelial growth factor in neurodegeneration and cognitive decline: exploring interactions with biomarkers of Alzheimer disease. JAMA Neurol. 72, 520–529. 10.1001/jamaneurol.2014.4761 25751166 PMC4428948

[B34] Hopkins M. E. Davis F. C. VanTieghem M. R. Whalen P. J. Bucci D. J. (2012). Differential effects of acute and regular physical exercise on cognition and affect. Neuroscience 215, 59–68. 10.1016/j.neuroscience.2012.04.056 22554780 PMC3374855

[B35] Hötting K. Reich B. Holzschneider K. Kauschke K. Schmidt T. Reer R.. (2012). Differential cognitive effects of cycling versus stretching/coordination training in middle-aged adults. Health Psychol. 31, 145–155. 10.1037/a0025371 21895371

[B36] Hötting K. Schickert N. Kaiser J. Röder B. Schmidt-Kassow M. (2016). The effects of acute physical exercise on memory, peripheral BDNF and cortisol in young adults. Neural Plasticity 2016, 1–12. 10.1155/2016/6860573 PMC4942640 27437149

[B39] Kraemer R. R. Durand R. J. Acevedo E. O. Johnson L. G. Kraemer G. R. Hebert E. P.. (2004). Rigorous running increases growth hormone and insulin-like growth factor-i without altering ghrelin. Exp. Biol. Med. (Maywood) 229, 240–246. 10.1177/153537020422900304 14988516

[B40] Kraus R. M. Stallings H. W. Yeager R. C. Gavin T. P. (2004). Circulating plasma VEGF response to exercise in sedentary and endurance-trained men. J. Appl. Physiol. (1985) 96, 1445–1450. 10.1152/japplphysiol.01031.2003 14660505

[B41] Kujach S. Olek R. A. Byun K. Suwabe K. Sitek E. J. Ziemann E.. (2020). Acute sprint interval exercise increases both cognitive functions and peripheral neurotrophic factors in humans: the possible involvement of lactate. Front. Neurosci. 13:1455. 10.3389/fnins.2019.01455 32038149 PMC6989590

[B42] Labban J. D. Etnier J. L. (2018). The effect of acute exercise on encoding and consolidation of long-term memory. J. Sport Exerc. Psychol. 40, 336–342. 10.1123/jsep.2018-0072 30541411

[B43] Landers-Ramos R. Q. Jenkins N. T. Spangenburg E. E. Hagberg J. M. Prior S. J. (2014). Circulating angiogenic and inflammatory cytokine responses to acute aerobic exercise in trained and sedentary young men. Eur. J. Appl. Physiol. 114, 1377–1384. 10.1007/s00421-014-2861-6 24643426 PMC4048778

[B44] Lanz T. A. Bove S. E. Pilsmaker C. D. Mariga A. Drummond E. M. Cadelina G. W.. (2012). Robust changes in expression of brain-derived neurotrophic factor (BDNF) mRNA and protein across the brain do not translate to detectable changes in BDNF levels in CSF or plasma. Biomarkers 17, 524–531. 10.3109/1354750X.2012.694476 22672085

[B46] Laukkanen M. B. R. Held T. (1999). Beurteilung der Ausdauer aufgrund der VO2max: standards des BASPO. Schweizerische Zeitschrift Für Sportmedizin Und Sporttraumatologie 47, 173–174.

[B47] Lenth R. (2019). Package ‘emmeans’. Available online at: https://CRAN.R-project.org/package=emmeans.

[B103] Lüdecke D. Ben-Shachar M. Patil I. Makowski D. (2020). Extracting, Computing and Exploring the Parameters of Statistical Models using R. J. Open Source Softw. 5:2445. 10.21105/joss.02445

[B48] Mateo I. Llorca J. Infante J. Rodríguez-Rodríguez E. Fernández-Viadero C. Peña N.. (2007). Low serum VEGF levels are associated with Alzheimer’s disease. Acta Neurol. Scand. 116, 56–58. 10.1111/j.1600-0404.2006.00775.x 17587256

[B104] MATLAB (2016). Version 9.1 (R2016b). Natick, MA: The MathWorks Inc.

[B49] McGaugh J. L. (1966). Time-dependent processes in memory storage. Science 153, 1351–1358. 10.1126/science.153.3742.1351 5917768

[B50] McGaugh J. L. (2000). Memory-a century of consolidation. Science 287, 248–251. 10.1126/science.287.5451.248 10634773

[B51] McNerney M. W. Radvansky G. A. (2015). Mind racing: the influence of exercise on long-term memory consolidation. Memory 23, 1140–1151. 10.1080/09658211.2014.962545 25312348

[B106] Meyer T. D. Hautzinger M. (2001). Allgemeine Depressions-Skala (ADS). Diagnostica 47, 208–215. 10.1026//0012-1924.47.4.208

[B52] Miranda M. Morici J. F. Zanoni M. B. Bekinschtein P. (2019). Brain-derived neurotrophic factor: a key molecule for memory in the healthy and the pathological brain. Front. Cell. Neurosci. 13:363. 10.3389/fncel.2019.00363 31440144 PMC6692714

[B53] Nader K. Hardt O. (2009). A single standard for memory: the case for reconsolidation. Nat. Rev. Neurosci. 10, 224–234. 10.1038/nrn2590 19229241

[B54] Niemann C. Godde B. Voelcker-Rehage C. (2014). Not only cardiovascular, but also coordinative exercise increases hippocampal volume in older adults. Front. Aging Neurosci. 6:170. 10.3389/fnagi.2014.00170 25165446 PMC4131191

[B55] Nishijima T. Piriz J. Duflot S. Fernandez A. M. Gaitan G. Gomez-Pinedo U.. (2010). Neuronal activity drives localized blood-brain-barrier transport of serum insulin-like growth factor-I into the CNS. Neuron 67, 834–846. 10.1016/j.neuron.2010.08.007 20826314

[B56] Pan W. Banks W. A. Fasold M. B. Bluth J. Kastin A. J. (1998). Transport of brain-derived neurotrophic factor across the blood-brain barrier. Neuropharmacology 37, 1553–1561. 10.1016/s0028-3908(98)00141-5 9886678

[B57] Pereira A. C. Huddleston D. E. Brickman A. M. Sosunov A. A. Hen R. McKhann G. M.. (2007). An *in vivo* correlate of exercise-induced neurogenesis in the adult dentate gyrus. Proc. Natl. Acad. Sci. 104, 5638–5643. 10.1073/pnas.0611721104 17374720 PMC1838482

[B58] Piccinni A. Marazziti D. Del Debbio A. Bianchi C. Roncaglia I. Mannari C.. (2008). Diurnal variation of plasma brain-derived neurotrophic factor (BDNF) in humans: an analysis of sex differences. Chronobiol. Int. 25, 819–826. 10.1080/07420520802387773 18780207

[B59] Prince M. Wimo A. Guerchet M. Ali G.-C. Wu Y.-T. Prina M. (2015). World Alzheimer Report. Available online at: https://www.alz.co.uk/research/WorldAlzheimerReport2015.pdf.

[B105] R Core Team (2018). R: A Language and Environment for Statistical Computing. Available online at: https://www.r-project.org.

[B60] Rich B. Scadeng M. Yamaguchi M. Wagner P. D. Breen E. C. (2017). Skeletal myofiber vascular endothelial growth factor is required for the exercise training-induced increase in dentate gyrus neuronal precursor cells. J. Physiol. 595, 5931–5943. 10.1113/JP273994 28597506 PMC5577548

[B61] Röder B. Demuth L. Streb J. Rösler F. (2003). Semantic and morpho-syntactic priming in auditory word recognition in congenitally blind adults. Lang. Cogn. Process. 18, 1–20. 10.1080/01690960143000407

[B62] Roig M. Nordbrandt S. Geertsen S. S. Nielsen J. B. (2013). The effects of cardiovascular exercise on human memory: a review with meta-analysis. Neurosci. Biobehav. Rev. 37, 1645–1666. 10.1016/j.neubiorev.2013.06.012 23806438

[B63] Roig M. Skriver K. Lundbye-Jensen J. Kiens B. Nielsen J. B. (2012). A single bout of exercise improves motor memory. PLoS One 7:e44594. 10.1371/journal.pone.0044594 22973462 PMC3433433

[B64] Roig M. Thomas R. Mang C. S. Snow N. J. Ostadan F. Boyd L. A.. (2016). Time-dependent effects of cardiovascular exercise on memory. Exerc. Sport Sci. Rev. 44, 81–88. 10.1249/JES.0000000000000078 26872291

[B65] Schmidt-Kassow M. Zink N. Mock J. Thiel C. M. Vogt L. Abel C.. (2014). Treadmill walking during vocabulary encoding improves verbal long-term memory. Behav. Brain Funct. 10:24. 10.1186/1744-9081-10-24 25015595 PMC4114134

[B66] Segal S. K. Cotman C. W. Cahill L. F. (2012). Exercise-induced noradrenergic activation enhances memory consolidation in both normal aging and patients with amnestic mild cognitive impairment. J. Alzheimers Dis. 32, 1011–1018. 10.3233/JAD-2012-121078 22914593 PMC3951984

[B67] Siette J. Reichelt A. C. Westbrook R. F. (2014). A bout of voluntary running enhances context conditioned fear, its extinction and its reconsolidation. Learn. Mem. 21, 73–81. 10.1101/lm.032557.113 24429425 PMC3895230

[B68] Skriver K. Roig M. Lundbye-Jensen J. Pingel J. Helge J. W. Kiens B.. (2014). Acute exercise improves motor memory: exploring potential biomarkers. Neurobiol. Learn. Mem. 116, 46–58. 10.1016/j.nlm.2014.08.004 25128877

[B69] Statton M. A. Encarnacion M. Celnik P. Bastian A. J. (2015). A single bout of moderate aerobic exercise improves motor skill acquisition. PLoS One 10:e0141393. 10.1371/journal.pone.0141393 26506413 PMC4624775

[B70] Stroth S. Hille K. Spitzer M. Reinhardt R. (2009). Aerobic endurance exercise benefits memory and affect in young adults. Neuropsychol. Rehabil. 19, 223–243. 10.1080/09602010802091183 18609015

[B71] Suwabe K. Hyodo K. Byun K. Ochi G. Yassa M. A. Soya H. (2017). Acute moderate exercise improves mnemonic discrimination in young adults. Hippocampus 27, 229–234. 10.1002/hipo.22695 27997992 PMC5927776

[B73] Thomas R. Beck M. M. Lind R. R. Korsgaard Johnsen L. Geertsen S. S. Christiansen L.. (2016a). Acute exercise and motor memory consolidation: the role of exercise timing. Neural Plast. 2016:6205452. 10.1155/2016/6205452 27446616 PMC4947505

[B74] Thomas R. Johnsen L. K. Geertsen S. S. Christiansen L. Ritz C. Roig M.. (2016b). Acute exercise and motor memory consolidation: the role of exercise intensity. PLoS One 11:e0159589. 10.1371/journal.pone.0159589 27454423 PMC4959698

[B72] Thomas A. G. Dennis A. Rawlings N. B. Stagg C. J. Matthews L. Morris M.. (2016). Multi-modal characterization of rapid anterior hippocampal volume increase associated with aerobic exercise. Neuroimage 131, 162–170. 10.1016/j.neuroimage.2015.10.090 26654786 PMC4848119

[B75] Tsai C.-L. Ukropec J. Ukropcová B. Pai M.-C. C. (2018). An acute bout of aerobic or strength exercise specifically modifies circulating exerkine levels and neurocognitive functions in elderly individuals with mild cognitive impairment. Neuroimage Clin. 17, 272–284. 10.1016/j.nicl.2017.10.028 29527475 PMC5842646

[B76] United Nations (2013). World Population Ageing 2013. United Nations.

[B77] Uysal N. Kiray M. Sisman A. Camsari U. Gencoglu C. Baykara B.. (2015). Effects of voluntary and involuntary exercise on cognitive functions and VEGF and BDNF levels in adolescent rats. Biotech. Histochem. 90, 55–68. 10.3109/10520295.2014.946968 25203492

[B78] van Dongen E. V. Kersten I. H. P. Wagner I. C. Morris R. G. M. Fernández G. (2016). Physical exercise performed four hours after learning improves memory retention and increases hippocampal pattern similarity during retrieval. Curr. Biol. 26, 1722–1727. 10.1016/j.cub.2016.04.071 27321998

[B79] van Praag H. (2008). Neurogenesis and exercise: past and future directions. Neuromolecular Med. 10, 128–140. 10.1007/s12017-008-8028-z 18286389

[B80] Vaynman S. Ying Z. Gomez-Pinilla F. (2004). Hippocampal BDNF mediates the efficacy of exercise on synaptic plasticity and cognition. Eur. J. Neurosci. 20, 2580–2590. 10.1111/j.1460-9568.2004.03720.x 15548201

[B81] Vogel S. Schwabe L. Schachinger H. Oitzl M. S. Zmyj N. (2016). Learning and memory under stress: implications for the classroom. NPJ Sci. Learn. 1:16011. 10.1038/npjscilearn.2016.11 30792896 PMC6380371

[B82] Voss M. W. Vivar C. Kramer A. F. van Praag H. (2013). Bridging animal and human models of exercise-induced brain plasticity. Trends Cogn. Sci. 17, 525–544. 10.1016/j.tics.2013.08.001 24029446 PMC4565723

[B83] Webb N. J. A. Bottomley M. J. Watson C. J. Brenchley P. E. C. (1998). Vascular endothelial growth factor (VEGF) is released from platelets during blood clotting: implications for measurement of circulating VEGF levels in clinical disease. Clin. Sci. (Lond) 94, 395–404. 10.1042/cs0940395 9640345

[B84] Winter B. Breitenstein C. Mooren F. C. Voelker K. Fobker M. Lechtermann A.. (2007). High impact running improves learning. Neurobiol. Learn. Mem. 87, 597–609. 10.1016/j.nlm.2006.11.003 17185007

[B85] Woost L. Bazin P.-L. Taubert M. Trampel R. Tardif C. L. Garthe A.. (2018). Physical exercise and spatial training: a longitudinal study of effects on cognition, growth factors and hippocampal plasticity. Sci. Rep. 8:4239. 10.1038/s41598-018-19993-9 29523857 PMC5844866

[B86] World Medical Association (2013). World Medical Association Declaration of Helsinki: ethical principles for medical research involving human subjects. JAMA 310, 2191–2194. 10.1001/jama.2013.281053 24141714

